# Breastfeeding in Preterm Infants Is Not Compromised by Early Discharge and Home Nasogastric Tube Feeding up to 3 Months Postmenstrual Age: A Prospective Cohort Study

**DOI:** 10.3390/nu17152444

**Published:** 2025-07-26

**Authors:** Rahel Schuler, Alice Louise Kreidler, Markus Waitz, Birgit Kampschulte, Jutta Petzinger, Tina Frodermann, Andreas Hahn, Walter A. Mihatsch

**Affiliations:** 1 Department of General Pediatrics and Neonatology, Justus-Liebig-University, Feulgenstrasse 12, D-35392 Giessen, Germany; alice.l.kreidler@med.uni-giessen.de (A.L.K.); markus.waitz@gnh.net (M.W.); birgit.kampschulte@paediat.med.uni-giessen.de (B.K.); jutta.petzinger@paediat.med.uni-giessen.de (J.P.); tina.lauer@paediat.med.uni-giessen.de (T.F.); 2 Department of Pediatric Neurology, Justus-Liebig-University, Feulgenstrasse 12, D-35392 Giessen, Germany; andreas.hahn@paediat.med.uni-giessen.de; 3 Division of Neonatology and Pediatric Intensive Care Medicine, Department of Pediatrics and Adolescent Medicine, University Medical Center Ulm, Eythstr. 24, D-89075 Ulm, Germany; walter.mihatsch@uni-ulm.de; 4 Department Pediatrics, Balingen Pediatric Hospital, Tuebinger Str. 31, D-72336 Balingen, Germany

**Keywords:** early discharge, family-centered care, breastmilk, breastfeeding, preterm

## Abstract

**Background/Objectives**: Breastmilk offers numerous benefits for the health and development of preterm infants, while prolonged hospitalization may impair neurodevelopment. At our institution, the implementation of enhanced family-centered care (FCC) has enabled earlier discharge of preterm infants. This study aimed to assess the impact of early discharge on breastfeeding and breastmilk provision. **Methods**: This analysis is based on data from a prospective single-center longitudinal cohort study conducted from October 2020 to November 2023, involving six consecutive cohorts (one baseline and five intervention cohorts; n = 184). FCC was progressively enhanced across cohorts. The primary outcome of the main study was postmenstrual age (PMA) at discharge. In this secondary analysis, breastfeeding and breastmilk provision were assessed at four time points: 4 weeks postnatal age, at discharge, 4 weeks post-discharge, and at 3 months PMA. **Results**: From baseline to intervention cohort 5, the PMA at discharge declined significantly from 37.8 ± 2.1 to 35.7 ± 0.91 weeks (*p* = 0.03), while the percentage of infants necessitating home nasogastric tube feeding increased from 6.3% to 66.7% (*p* < 0.01). The proportion of breastmilk of daily feeding volume remained unchanged at 4 weeks postnatal age (0.66 ± 0.42 vs. 0.9 ± 0.28) and at discharge (0.6 ± 0.45 vs. 0.79 ± 0.36). At 4 weeks post-discharge, 65.8% vs. 62.5% of the infants were on partial or exclusive breastmilk (*p* = 0.91) feeding. Similarly, the percentage of exclusively breastfed infants at 4 weeks post-discharge (23.7% vs. 19.8%) and at 3 months PMA (20% vs. 28.6%) did not differ significantly between baseline and intervention cohort 5. **Conclusions**: Early discharge did not reduce breastmilk supply or exclusive breastfeeding. However, the persistently low rate of exclusive breastfeeding post-discharge highlights the need for additional support strategies during and after hospitalization.

## 1. Introduction

Breastmilk provides nutritional, immunological, and neurodevelopmental benefits [1]. For example, a recent review reported a modest, dose-dependent association between breastfeeding and improved cognitive outcomes [2]. In preterm infants, breastmilk feeding was associated with a lower incidence of short-term morbidities, such as Bronchopulmonary Dysplasia (BPD) [3], Necrotizing Enterocolitis (NEC) and sepsis [4], faster achievement of full enteral feeds, and a reduced number of days on antibiotics [4]. In addition, long-term benefits such as a reduced risk of suboptimal neurodevelopment at 3 and 5 years in preterm infants breastfed at discharge have been described [5]. Finally, breastfeeding helped preterm infants’ mothers to regain a sense of normality [6] and supports mother–infant bonding [7].

Breastfeeding practices are influenced by a range of clinical variables, such as postmenstrual age (PMA) at birth [8], the timepoint of first breastmilk expression [9], the initiation of skin-to-skin contact (SSC) [10,11], and mode of delivery [12].

Prolonged hospitalization has been associated with poorer neurodevelopmental outcomes in preterm infants, as demonstrated in two observational studies [13,14]. In one study, an early discharge cohort receiving home neonatal care showed significantly better neurodevelopmental outcomes compared to a large national French cohort discharged in 2011, even after adjustment for postmenstrual age at birth and neonatal morbidities [13]. Consequently, an increasing number of neonatal intensive care units (NICUs) are adopting early discharge programs. Previous studies found that early discharge did not increase unplanned readmissions [15] or parental anxiety [16] and was well-received by the parents [15,17].

The final weeks before discharge are often dedicated to providing intensive lactation support, including assistance with breastfeeding positioning, combining nasogastric tube feeding with direct breastfeeding, and determining the appropriate volume of breastmilk to administer via tube after a breastfeeding session. Earlier discharge may limit access to this individualized support, potentially affecting both the initiation and continuation of breastfeeding. Data on breastfeeding in very low birthweight (VLBW) infants after early discharge are sparse and conflicting. While some studies reported improved breastfeeding after early discharges [18,19], others did not find this association [15,16].

In the context of a prospective longitudinal cohort study on the gradual implementation of family-centered care (FCC), our institution has introduced enhanced parental involvement in the care of very preterm infants. This approach has been associated with earlier discharge—[20] approximately two weeks prior to the international average [21]. The present study aims to evaluate the potential impact of this earlier discharge on breast milk provision at discharge and up to three months PMA.

## 2. Materials and Methods

Study design: This study is part of an ongoing prospective longitudinal cohort study evaluating the impact of a stepwise implementation of FCC in a German perinatal level III center. This study is registered at ClinicalTrials.gov (NCT05286983) and received ethical approval from the local Institutional Review Board prior to initiation (approval number AZ 153/20) [22].

Eligible participants were preterm infants born at or before 32+0 weeks PMA and/or a birthweight ≤ 1500 g. Exclusion criteria included major congenital anomalies (e.g., cyanotic heart disease, severe pulmonary hypoplasia), decision for palliative care prior to enrolment, or severe parental psychiatric conditions (e.g., acute psychosis). Written informed consent was obtained from the parents prior to participation.

The primary outcome of this study is the length of hospital stay measured by PMA at discharge. In a previous analysis, we demonstrated a non-significant reduction in PMA at discharge across all preterm infants, with a significant decline observed in those without neonatal morbidities [20].

In the present study, we aim to evaluate the impact of this earlier discharge on breastmilk provision and breastfeeding, one of the predefined secondary outcomes. Our prespecified hypothesis was that earlier discharge would not adversely affect breastmilk provision or breastfeeding rates.

Breastmilk provision and/or breastfeeding were assessed at four different time points: at four weeks postnatal age (proportion of breastmilk), at discharge (proportion of breastmilk and number of patients receiving partial or exclusive breastmilk feeding), at four weeks post discharge (number of patients receiving breastmilk and number of patients partially or exclusively breastfed) and at three months PMA (number of patients exclusively breastfed).

Neonatal morbidities were defined as follows: bronchopulmonary dysplasia (BPD) according to the physiological definition by Walsh [23], intraventricular hemorrhage (IVH) ≥ grade III and PVL as diagnosed by ultrasound [24], retinopathy of prematurity (ROP) ≥ stage 3 or treatment of ROP, and necrotizing enterocolitis (NEC) ≥ Stage 2 [25].

**Figure 1:**
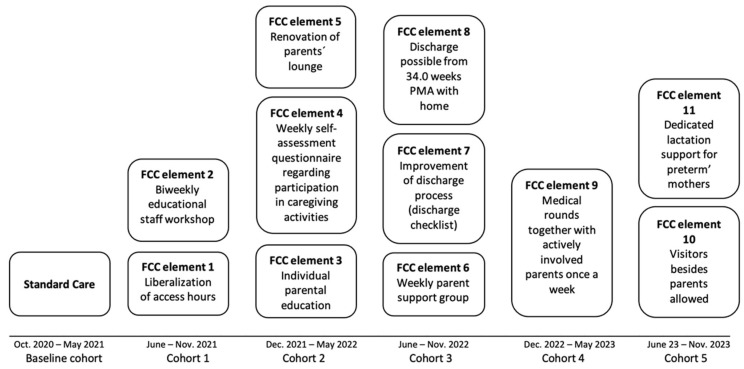
Timeline of FCC elements of the intervention.

Intervention: The baseline cohort (October 2020–May 2021) included 45 VLBW infants prior to FCC implementation. Discharge occurred following the establishment of full oral feeding. Five subsequent intervention cohorts consisted of infants and their parents recruited consecutively within 6-month periods from June 2021 to November 2023. Starting in June 2021, FCC elements were progressively introduced; these are described in Figure 1. With the enhancement of FCC and the education of parents to become the primary caregivers for their infant, the practice of discharge on full oral feeding gradually changed. Parents were taught to handle the nasogastric tube early on during the NICU stay, and discharge on nasogastric tube feeding became the standard. Mothers were encouraged to breastfeed from 32 weeks PMA, often in combination with nasogastric tube feeding.

The provision of breastmilk was strongly promoted throughout this study. The whole medical team attended a workshop on the importance of breastfeeding and practical steps to improve breastfeeding were implemented in the obstetric and neonatal wards thereafter. Prenatal and postnatal counselling on the importance of breastmilk for preterm infants, the availability of bedside pumps, and staff training in lactation support were key components of the initiative.

For the purpose of this study, ‘first skin-to-skin contact’ was defined as the earliest occasion on which the infant, unclothed except for a diaper, was placed directly on the bare chest of either parent. This procedure was consistently applied and remained unchanged throughout the study period.

Although the unit lacked single-family rooms (SFRs), rooming-in was available in the stepdown unit with two mothers or, less frequently, fathers sharing one room together with their infants.

Throughout this study, all families were offered neonatal home care upon discharge; however, participation was not mandatory. If parents opted to participate, a nurse or a social worker visited the family at least once a week for 12 weeks after discharge to monitor weight gain, support breastfeeding, answer questions, and assist with medical follow-ups.

The term “breastmilk” refers specifically to a mother’s own milk. The term “exclusive breastmilk” is defined as the exclusive provision of the mother’s own milk, regardless of the mode of feeding. The term “partial breastmilk” denotes feeding any combination of breastmilk and formula, again irrespective of the mode of feeding. The term “breastfed” refers to infants fed either exclusively or partially (accompanied by bottle or tube) at the mother’s breast. The term “exclusively breastfed” refers to feeding exclusively at the mother’s breast, without the use of bottles or a nasogastric tube.

Postmenstrual age (PMA) was calculated by adding chronological age at the time of assessment to the gestational age at birth, as per standard neonatal practice.

Data collection: Patient characteristics and feeding practice at four weeks after birth, at discharge, and at three months PMA were extracted from the hospital and outpatient department medical records. Post-discharge feeding practices at four weeks were evaluated through a self-administered questionnaire distributed to families; in cases where the questionnaire was not returned, data were subsequently obtained via telephone interview. Certified interpreters assisted non-German speakers. All data were collected prospectively, with the exception of feeding outcomes at 3 months PMA, which were retrospectively extracted from routine outpatient medical records.

Statistical analysis: Data analysis was conducted using SPSS version 28.9.1.1. (14) (IBM Corp., Armonk, New York, NY, USA). Data are presented as mean ± standard deviation (SD) (median; Q1–Q3) or as number (percentage) as appropriate. The relative proportion of breast milk in the daily milk intake is given as a decimal number (from 0.0 to 1.0), while the proportion of infants receiving a particular diet (e.g., exclusive or partial breastfeeding) is given as a percentage. The Fisher’s Exact Test was used for count data with simulated *p*-value (based on 2000 replicates) between the six cohorts (Table 1 and Table 2). The Kruskal–Wallis rank sum test, together with the post hoc Dunnett test comparing intervention cohort 5 with the baseline cohort, was used to compare continuous variables between the groups (Table 1). A *p*-value < 0.05 was considered statistically significant.

Study participants: Of 260 eligible infants, a total of 184 (70.1%) were enrolled. Thirteen families declined consent, 34 met exclusion criteria, 23 were not approached due to staffing shortage, and six infants died and were excluded from further analysis.

## 3. Results

Clinical data and complications during hospital stay are summarized in Table 1. PMA at discharge declined during this study from 37.8 (±2.1) to 35.7 (±0.91) weeks PMA (*p* = 0.03). Significantly, more infants were discharged on nasogastric tube feeding in the intervention cohort 5 (*p* < 0.01). Furthermore, significant differences between the single cohorts were found for in-hospital mortality and PMA at rooming-in.

There were no significant differences regarding breastmilk provision across all cohorts at any time point despite progressively earlier discharge (Table 2).

The average proportion of breastmilk at 4 weeks of age increased non-significantly across the study period from 0.66 (±0.42) at baseline to 0.90 (±0.28) in intervention cohort 5. At discharge, 88.9% of the infants in intervention cohort 5 were fed partially or completely with breastmilk, and the mean breastmilk proportion on the feeding volume was 0.79 (±0.36) (Table 2).

After discharge, the provision of breastmilk declined in all cohorts. However, at 4 weeks, 62.5% of the infants in intervention cohort 5 received some breastmilk, and 18.8% were exclusively breastfed (Table 2).

At three months, the exclusive breastfeeding rate remained stable across the cohorts, with no significant decline observed from the baseline to intervention cohort 5. Rates ranged from 17.4% to 34.8% of infants (Table 2).

## 4. Discussion

In this study, we describe the effects of an early discharge program on breastmilk provision in a larger cohort of very preterm infants up to three months PMA. Our findings indicate that early discharge with nasogastric tube feeding did not reduce breastmilk provision or rates of exclusive breastfeeding up to three months PMA, thereby supporting our prespecified hypothesis. However, given the multiple benefits of breastmilk for preterm infants, further improvement is needed. Comparison of our findings with the existing literature is limited by heterogeneity in postmenstrual age at birth, timing of outcome assessments, and the specific aspects of breastmilk provision analyzed across study populations [18,19,26,27].

During the hospital stay, the proportion of breastmilk provided declined from four weeks of age until discharge in all cohorts. One possible explanation for this trend is pumping fatigue, as very preterm infants in our unit typically do not begin direct breastfeeding before 32 weeks PMA. Furthermore, the observed decline may suggest a need to strengthen parental education and support regarding the sustained importance and long-term benefits of breastmilk for preterm infants.

At discharge, more than 2/3 of all study infants received some breastmilk, and nearly half of the infants were on exclusive breastmilk feeding. This is in principal agreement with other European countries, although there is considerable variation for preterm infants <32 weeks across different regions and countries [7,28]. For example, in a recent study involving preterm infants with a mean PMA at birth of 28 weeks, only 50% received some breastmilk at the time of discharge [26], which was substantially less than in all cohorts of our study.

At four weeks after discharge, approximately 60% of all our study patients received partial breastmilk. This rate is lower than approximately 90% in two other early discharge studies at a similar time point [18,19]. However, the infants in these studies were considerably more mature at birth, and the odds of breastfeeding failure are inversely related to PMA at birth [29].

Generally, exclusive breastfeeding declines within the first months in term and preterm infants [19,30]. At three months, the percentage of mothers of term newborns providing exclusive breastfeeding dropped to 70% in an observational questionnaire-based study [30]. In Germany, 60% of all infants were exclusively breastfed at two months and 50% at four months [31]. In our study, the rate of exclusive breastfeeding was approximately 25% and did not change significantly from 4 weeks after discharge until 3 months PMA. The percentage of preterm infants exclusively fed breastmilk has been shown to decrease steadily from birth to six months post-discharge [19,32]. A prospective longitudinal study of 104 infants born at 32 weeks PMA investigating different aspects of Kangaroo Mother Care found that at 2 months PMA, 40% were exclusively breastfed [27]. In another prospective cohort study including 94 infants of 33 weeks PMA at birth, 33% were exclusively breastfed at three months PMA, which is similar to our final intervention cohort, although our infants were much more immature at birth [32]. Overall, breastfeeding rates at three months PMA in our cohort are consistent with findings from other studies.

Nonetheless, considering the advantages of breastmilk, continued strategies to enhance its availability and use remain essential. Several modifiable factors to improve breastmilk provision during the hospital stay have been described. Unit policies supporting breastmilk feeding were an effective intervention for improved breastmilk provision [11,33], while, surprisingly, the involvement of a professionally trained lactation consultant did not significantly enhance breastmilk provision [11].

Initiation of breastfeeding during the hospital stay was associated with breastmilk provision at discharge [10]. On the other hand, the physical separation from the infant, the stressful NICU surroundings, structured feeding routine, and lack of privacy hindered breastmilk provision [6].

While policies to support breastmilk feeding and to initiate breastfeeding during the hospital stay are routine in our NICU, interventions to facilitate privacy and closeness of the parents with their infant may need improvement.

The early initiation of SSC, particularly within the first week of life and immediately postpartum in the delivery room, has been associated with increased rates of breastmilk provision at the time of hospital discharge [10,11,34]. In our cohorts, SSC was initiated later, between the third and fourth day of life, in contrast to earlier initiation reported by other centers [27,29]. For instance, one study reported a median initiation time of 6 h after birth, with the latest initiation observed at 78 h [27]. In a pertinent survey on lactation support in German NICUs, only 5% of preterm infants’ mothers were able to have SSC in the delivery room, and only 62% within the first day of life [35]. Parents themselves experienced holding their infant as a facilitator for breastfeeding [6]. Taken together, these findings suggest that earlier initiation of SSC in our NICU may potentially enhance breast milk provision.

In addition, earlier first breastmilk expression may improve breastmilk provision [9,29]. In a German study, only 36% of mothers pumped within 6 h after birth, and only half within 24 h [35]. Communication issues between professionals in the maternity ward and NICU were described as barriers [35]. Unfortunately, we did not record the time point of first breastmilk expression. Communication issues between the wards were regularly mentioned by parents in our NICU, and measures for improvement have been taken.

Given that cesarean delivery has been linked to reduced breastfeeding rates in term infants [36], the high proportion of cesarean births in our cohort may have contributed to the low exclusive breastfeeding rates observed at three months PMA.

Family-integrated care is a concept that allows parents to be the primary caregivers of their infant and actively participate in their care [37]. This concept was associated with improved breastfeeding outcomes during a hospital stay up to discharge [37,38]. In our study, the enhancement of parental involvement [20] did not result in an increase in breastmilk provision at discharge, although it was high throughout this study. This is in line with the first cluster-randomized family-integrated care trial, which did not result in higher breastmilk provision at discharge, while infants in the intervention group were more frequently breastfed within 24 h [38].

Infants cared for in single-family rooms (SFRs) have been shown to have higher rates of exclusive breastfeeding up to four months PMA. At four months PMA, approximately 15% of these infants were still exclusively breastfed [39]. However, this proportion was notably lower than the rates observed across all cohorts in our study at three months PMA.

Our unit does not provide SFRs in the NICU, and rooming-in is possible in the stepdown unit. Moving in with the infant was initiated at a steadily decreasing PMA from the baseline cohort to intervention cohort 5, which did not significantly improve breastfeeding up to discharge or thereafter.

There is evidence that longer hospital stays are negatively associated with breastfeeding at discharge [40], and by contrast, early discharge with neonatal home care was associated with improved breastfeeding rates up to 6 months [18,19,41]. In two studies, preterm infants in the early discharge group had a higher rate of breastfeeding or breast milk intake at discharge from the neonatal home care program compared to the standard care group [18,41]. Additionally, one study found a significantly higher proportion of exclusively breastfed preterm infants up to six months [19]. These findings suggest that early discharge supported by neonatal home care may contribute to prolonged breastfeeding. However, while not all early discharge programs have demonstrated this benefit, none have reported a negative impact on breastfeeding outcomes [15,16,42]. In our study, significantly earlier discharge with neonatal home care in intervention cohort 5 did not result in improved breastmilk provision or breastfeeding.

Altogether, these data show that early discharge with neonatal home care did not consistently result in longer breastfeeding, and therefore is not sufficient to sustain breastfeeding in preterm infants. It remains unclear whether the observed variations in breastfeeding outcomes are attributable to differences in the structure and content of neonatal home care programs, as this aspect has not yet been systematically investigated. In addition to in-hospital interventions aimed at promoting breastfeeding, targeted measures implemented during neonatal home care may also contribute to prolonged breastfeeding duration. However, these potential effects have not been explored to date.

Our study has several limitations. As this was a single-center study with a small sample size, our results cannot easily be transferred to other neonatal units. Due to the longitudinal cohort design, confounding factors over time cannot be entirely excluded. The number of infants in the final cohort was relatively small, which may limit the generalizability of the findings. Additionally, missing data on post-discharge feeding practices resulted from loss to follow-up. This also applies to the four-week postnatal time point, as some infants had been discharged by that age. Feeding data at four weeks post-discharge were obtained through self-reported questionnaires, introducing the potential for recall and reporting bias. In addition, we only retrospectively collected data on exclusive breastfeeding at three months PMA and do not have data on other breastmilk outcomes at this point in time.

## 5. Conclusions

Our findings suggest that the stepwise enhancement of FCC, resulting in earlier discharge, was not associated with adverse effects on breastfeeding outcomes up to three months PMA. However, given the well-established benefits of breastmilk—particularly for preterm infants—there remains a need for targeted interventions to support and promote breastfeeding, especially in the context of neonatal home care. Future research should focus on identifying effective strategies to extend breastfeeding duration following early discharge.

## Figures and Tables

**Table 1:** Patient characteristics of all cohorts during hospital stay.

	Baseline Cohort	Cohort 1	Cohort 2	Cohort 3	Cohort 4	Cohort 5	*p*-Value **
n	51	35	26	27	30	21	
PMA at birth, weeks	28.3 ± 3.0 (29.0; 25.6–30.9)	29.2 ± 2.7 (29.4; 27.9–31.4)	27.9 ± 2.8 (28.1; 26.1–29.6)	29.3 ± 2.8 (29.6; 27.9–31.4)	28.1 ± 2.9 (29.4; 26.0–30.4)	28.3 ± 2.8 (28.5; 26.0–30.6)	0.34
Outborn, n (%)	2 (3.9%)	0	0	0	0	0	0.65
In hospital death, n (%)	3 (5.9%)	0	0	0	0	3 ^#1^ (14%)	0.027
Survival till discharge	48	35	26	27	30	18	
Caesarean section n (%)	43 (89.6%)	35 (100.0%)	26 (100.0%)	26 (96.3%)	29 (96.7%)	18 (100%)	0.16
Male sex, n (%)	18 (37.5%)	18 (51.4%)	15 (57.7%)	12 (44.4%)	16 (53.3%)	11 (61.1%)	0.43
PMA at birth, weeks	28.5 ± 2.9 (29.3; 25.8–31.0)	29.2 ± 2.7 (29.4; 27.9–31.4)	27.9 ± 2.8 (28.1; 26.1–29.6)	29.3 ± 2.8 (29.6; 27.9–31.4)	28.1 ± 2.9 (29.4; 26.0–30.4)	29.1 ± 2.2 (29.4; 27.9–30.6)	0.37
Birthweight, g	1088 ± 394 (1115; 730–1435)	1174 ± 414 (1240; 850–1400)	1006 ± 377 (985; 730–1300)	1261 ± 387 (1360; 940–1555)	1069 ± 341 (1030; 850–1.310)	1193 ± 315 (1205; 975–1450)	0.12
Multiples, n (%)	17 (35%)	6 (17%)	8 (30.8%)	10 (37.0%)	9 (30%)	2 (11%)	0.21
Any neonatal morbidity *, n (%)	8 (17%)	6 (17%)	6 (23%)	2 (7%)	3 (10%)	3 (17%)	0.66
First skin-to-skin contact, day of life	3.85 ± 2.84 (3; 2–6)	3.51 ± 3.72 (3; 1–4)	4.88 ± 3.83 (4; 3–6)	3.30 ± 2.61 (3; 1–6)	3.47 ± 3.17 (3; 1–5)	3.17 ± 2.18 (3; 2–5)	0.42
PMA at rooming in, weeks	36.3 ± 2.4 (35.9; 34.9–36.4)	36.9 ± 2.7 (36.3; 35.3–37.6)	35.4 ± 2.2 (34.4; 34.1–35.9)	35.3 ± 1.4 (35.4; 34.9–36.6)	33.9 ± 1.7 (34.0; 32.9–35.2)	34.46 ^#2^ ± 1.65 (34.50; 33.07–35.86)	<0.001
PMA at discharge, weeks	37.8 ± 2.1 (37.6; 36.4–38.7)	37.5 ± 2.9 (36.6; 35.6–38.1)	37.8 ± 4.0 (36.4; 35.6–39.3)	36.9 ± 2.8 (36.4; 35.1–37.9)	36.4 ± 1.9 (35.8; 35.1–37.3)	35.7 ^#3^ ± 0.9 (35.7; 35.1–36.4)	0.001
Nasogastric tube feeding at discharge, n (%)	3 (6.3%)	4 (11%)	12 (46%)	13 (48%)	22 (73%)	12 ^#4^ (67%)	<0.001
Neonatal home care, n (%)	38 (79%)	22 (63%)	21 (81%)	19 (70%)	25 (83%)	15 (83%)	0.37

PMA = Postmenstrual Age, * Any Neonatal Morbidity = Bronchopulmonary Dysplasia or Intraventricular Hemorrhage or Periventricular Leukomalacia or Retinopathy of Prematurity or Necrotizing Enterocolitis or Focal Intestinal Perforation, data is given as n (%) or mean ± SD (median; Q1–Q3) as appropriate; ** Kruskal–Wallis or Fisher’s exact test as appropriate; post hoc Dunnett test, ^#1^ <0.24; ^#2^ <0.15; ^#3^ <0.02; ^#4^ <0.001).

**Table 2:** Nutrition of all cohorts during hospital stay and until 3 months PMA.

	Baseline Cohort	Cohort 1	Cohort 2	Cohort 3	Cohort 4	Cohort 5	*p*-Value **
Nutrition at 4 weeks postnatal age							
Number of infants, N	46°	33°	25°	23°	27°	16°	
Proportion of Breastmilk (SD)	0.66 ± 0.4 (1.0; 0.3–1.0)	0.75 ± 0.4 (1.0; 0.6–1.0)	0.75 ± 0.4 (1.0; 0.6–1.0)	0.76 ± 0.4 (1.0; 0.5–1.0)	0.82 ± 0.4 (1.0; 0.9–1.0)	0.9 ± 0.3 (1.0; 1.0–1.0)	0.40
Nutrition at discharge							
Number of infants, N	48	35	26	27	30	18	
Proportion of breastmilk (SD)	0.6 ± 0.5 (0.9; 0.0–1.0)	0.61 ± 0.5 (0.8; 0.0–1.0)	0.55 ± 0.4 (0.67; 0.0–1.0)	0.56 ± 0.5 (0.8; 0.0–1.0)	0.6 ± 0.5 (0.9; 0.0–1.0)	0.79 ± 0.4 (1.0; 0.5–1.0)	0.40
Partial or exclusive breastmilk, n (%)	35 (72.9%)	25 (71.4%)	19 (73.1%)	18 (66.7%)	21 (70.0%)	16 (88.9%)	0.71
Exclusive breastmilk, n (%)	23 (47.9%)	16 (45.7%)	8 (30.8%)	10 (37.0%)	13 (43.3%)	12 (66.7%)	0.28
**Nutrition at 4 weeks after discharge**							
Number of infants, N	38	30	20	23	28	16	
Partial or exclusive breastmilk, n (%)	25 (65.8%)	17 (56.7%)	14 (70%)	14 (60.9%)	15 (55.6%)	10 (62.5%)	0.92
Exclusive breastmilk, n (%)	11/37 * (29.7%)	10 (33.3%)	8 (40.0%)	7 (30.4%)	11 (40.7%)	7 (43.8%)	0.87
Breastfed, n (%)	20 (52.6%)	13/29 * (44.8%)	11 (55.0%)	12 (52.2%)	13 (46.4%)	8 (50.0%)	0.98
Exclusively breastfed, n (%)	9 (23.7%)	8/29 * (27.6%)	5/18 * (27.8%)	4/22 * (18.2%)	4/25 * (16.9%)	3 (18.8%)	0.91
**Nutrition at 3 months PMA**							
Number of infants, N	45	31	23	23	26	17	
Exclusively breastfed n (%)	9 (20%)	6 (19.4%)	8 (34.8%)	4 (17.4%)	6 (23.1%)	4 (23.3)	0.77

PMA = Postmenstrual Age, ° missing data due to discharge before 4 weeks postnatal age, * missing answers on questionnaire, ** Kruskal–Wallis or Fisher’s exact test as appropriate. The table displays two types of data: (1) continuous proportions (ranging from 0.1 to 1.0), indicating the individual proportion of breastmilk in each infant’s total nutritional intake at specified timepoints; and (2) categorical percentages, representing the proportion or rate of infants receiving breast milk according to different exposure categories (e.g., exclusive breastfeeding, partial breastfeeding) at the corresponding timepoints.

## Data Availability

The raw data supporting the conclusions of this article will be made available by the authors on request. For privacy reasons the data are not public.

## Abbreviations

The following abbreviations are used in this manuscript:

BPD	Bronchopulmonary Dysplasia
FCC	Family-Centered Care
IVH	Intraventricular Hemorrhage
NEC	Necrotizing Enterocolitis
NICU	Neonatal Intensive Care Unit
PMA	Postmenstrual Age
ROP	Retinopathy of Prematurity
SD	Standard Deviation
SFR	Single-Family Room
SSC	Skin-to-skin contact
VLBW	Very Low Birthweight
